# Effect of Nano-SiO_2_ on Expression and Aberrant Methylation of Imprinted Genes in Lung and Testis

**DOI:** 10.1186/s11671-018-2673-4

**Published:** 2018-09-04

**Authors:** Beilei Yuan, Huazhong Zhang, Xuan Wang, Yong Pan, Juncheng Jiang

**Affiliations:** 1 College of Safety Science and Engineering, Nanjing Tech University, 200 Zhongshan North Rd, Nanjing, 210009 China; 2 Department of Emergency Medicine, The First Affiliated Hospital of Nanjing Medical University, Nanjing, Jiangsu China

**Keywords:** Nanoparticle, Lung, Testis, Methylation

## Abstract

Nanotechnology has been developing rapidly and is now used in many cutting-edge medical therapeutics. However, there is increasing concern that exposure to nanoparticles (NPs) may induce different systemic diseases as epigenetic mechanisms are associated with more and more disease. The role of NP epigenomic modification is important to disease etiology. Our study aimed to determine the epigenetic mechanisms of damage in lung and testis cells by exposing cells to SiO_2_ NPs. We used male C57BL/6 mice to characterize the damaging effect of SiO_2_ NPs on lung and testis cells as well as the resulting methylation state at the imprinted Dlk1/Dio3 domain region. The A549 cells exposed to SiO_2_ NPs had cell apoptosis, and male mice exposed to SiO_2_ NPs had altered lung and testis tissues. The genes in the imprinted domains Dlk1/Dio3 region changed in both tissues; *Dlk1*, *Rtl1*, and *Dio3* are upregulated in testis while *Dlk1* and *Dio3* are also upregulated in lung tissues. Bisulfite sequencing PCR of male adult lung and testis were mostly hypomethylated, with a few hypermethylated CpGs. These findings indicate that nanoparticles play an important role in DNA methylation of imprinted genes.

**Electronic supplementary material:**

The online version of this article (10.1186/s11671-018-2673-4) contains supplementary material, which is available to authorized users.

## Background

Silicon dioxide is an oxide of silicon with the chemical formula SiO_2_, most commonly found in nature as quartz and in various organic environments [1]. Engineered nanoparticles have been widespread in the rapid growth and application of nanotechnology in high-tech industries. This particular nanoparticle is widely used in a range of consumer products including electronics, plastic products, medical, cosmetic, and coating material due to their physical scientific properties such as large specific surface area, abundant reactive sites, high surface energy, unsaturated chemical bonds, strong adsorption capability, and a strong tendency to interact with metals and organic matter, thereby altering contaminants and their transport in the environment [2]. The presence of SiO_2_ nanoparticles (NPs) in a wide range of consumables increases their likelihood of being released in the environment and comes into contact with the human population.

Previous experimental studies have shown that a single-dose intratracheal instillation or multiple intraperitoneal injections of a metal and metal-oxide nanoparticle species cause toxic effects from cellular to systemic and organismic levels [3]. Treatment of SiO_2_ NPs represses the growth of breast cancer cell lines by increasing apoptosis and reducing cell motility. Moreover, exposure to SiO_2_ NPs significantly disturbs the epidermal growth factor receptor (EGFR) [4]. When rat models were treated with three different sizes of TiO_2_ NPs and compared with controls, bronchoalveolar lavage fluid (BALF) treatment with large agglomerate (> 100 nm) aerosols induced an acute inflammatory response, while small agglomerate (< 100 nm) aerosols produced significant oxidative stress damage and cytotoxicity [5].

The study of nanoparticle toxicity on reproduction is a growing field. One study has demonstrated that under the same treatment dose, Ni NPs induced higher reproductive toxicity in *C. elegans* than Ni MPs (microparticles). These reproductive toxicities observed in *C. elegans* included reduced brood size, fertilized egg, and spermatide activation [6]. There is growing evidence that certain environmental effects can be passed to offspring via paternally pathways without changes in the sperm genome [7, 8]. Paternal information exists not only in the genome, but also in related specific epigenetic markers, mRNA content, and non-coding RNA.

Oxidative stress is an important mechanism in nanoparticle toxicity, which can trigger DNA damage, inflammation, protein denaturation, and lipid peroxidation [9]. These biological effects are influenced by the physiochemical properties of nanoparticles, including their size, surface area, shape, surface chemistry, functionalization, and solubility [10, 11]. There is growing evidence that clearly demonstrate exposure to nanoparticles may trigger epigenetic alterations in tissues and cells even at low, non-cytotoxic doses [12, 13]. Epigenetics is the study of heritable changes in gene function that do not involve changes in the DNA sequence including methylation of DNA, gene imprinting, histone modifications, and regulation by non-coding RNAs [14]. Such epigenetic alterations are associated with the development and progression of numerous pathological states and diseases [15]. Therefore, epigenetic effects are a crucial part of patient risk assessment screening at the cellular level.

The Dlk1/Dio3 imprinted domain contains three known differentially methylated regions (DMRs) that are paternally methylated: intergenic DMR (IG-DMR), maternally expressed 3-DMR (Gtl2-DMR), and Dlk1-DMR [16]. Previous studies suggest that the IG-DMR dictates the allelic methylation status of the *Gtl2* promoter DMR, which then controls gene expression across the entire cluster [17]. The mouse genome has a large number of imprinted genes at the Dlk1/Dio3 domain in the distal region of chromosome 12. The IG-DMR located between imprinted gene *Dlk1* and *Gtl2* is specifically methylated in the male germline and regulates the parental allele-specific expression of the imprinted gene region [18]. The IG-DMR methylation status is established before birth and is thus maintained throughout a male’s lifetime in the male germline during male germ-cell differentiation, meaning IG-DMR methylation is maintained in spermatogonia and spermatocytes of mature testis.

Our aim was to find the changes in male germline gene expression during spermatogenesis prior to transcriptional and translational silencing in order to explain the paternal influence on offspring through the environmental changes. Environmental factors can modify sperm transcriptional modifications, which can lead to alterations in progeny development. To carry out this investigation in our work, we used cell lines and mice as models for screening of the toxic effects of SiO_2_ NPs. To our knowledge, this is the first study demonstrating the epigenetic mechanisms of the Dlk1/Dio3 imprinted regions that nanoparticles cause damage in both lung and testis tissue.

## Methods

### Experimental Animal

Animal handling was performed in accordance with the Guide for the Care and Use of Laboratory Animals under the corresponding animal use protocol at the Nanjing Medical University. Mice were obtained from Beijing Vital River Laboratory Animal Technology Co., Ltd. All animals were housed at 23 °C with a 12-h light cycle. Sterilized water and rodent chow were consumed by the mice at will. Mice activity and behavior were monitored daily. After 2 weeks, mice were injected nano-sized SiO_2_ 12.5 mg/kg.

### Chemicals

Nano-sized SiO_2_ (99.5% trace metal basis, particle size 10–20 nm) were obtained from Sigma-Aldrich Chemical Co. (St. Louis, MO, USA). Nanoparticles were suspended in RPMI 1640 Medium to create a stock solution, dispersed by ultrasonic vibration for 20 min, diluted to appropriate concentrations, and dispersed for another 20 min.

### Characteristics of SiO_2_ NPs

The size and zeta potential were recorded using a Malvern Zetasizer Nano ZSP.

### RNA Extraction and qRT-PCR

The lung and testis samples were flash-frozen in liquid nitrogen and then stored at 80 °C after about 4 h. We defrosted the samples before we extracted the samples.

The total RNA was isolated from the samples using 1 mL of TRIzol Reagent (Invitrogen Life Technologies Co, USA). The mixture was ultrasonicated at 80% power for 5 min, added 0.2 mL of chloroform, and next was centrifugalized at 12,000*g*/min at 4 °C for 15 min. Then, three steps of phenol/chloroform purification were added in order to get rid of proteins. Then, we used UV absorbance to measure RNA content and quality of each sample at 260 and 280 nm. The primer sequences of mRNAs are showed in Additional file 1: Table S1 and S2. qRT-PCR was carried out using the manufacturer’s instructions, as described previously [19]. Real-time PCR was carried out using SYBR Green (Vazyme). The PCR cycle was as follows: initial denaturation at 95 °C for 30 s, followed by 40 cycles of denaturation at 95 °C for 15 s, annealing at 60 °C for 15 s, and extension at 72 °C for 30 s. The amount of target genes was analyzed using the 2^-ΔCt method following normalization with β-actin.

### DNA Extraction, Bisulphite Treatment, and Bisulfite Sequencing PCR

DNA was isolated from testicular and lung tissues using a DNA kit (QIAamp DNA Mini Kit; Qiagen. No.51304; USA) following the manufacturer’s recommendations. Bisulfite conversion of 500 ng of all genomic DNA was achieved using a kit (EpiTect® Bisulfite kit; Qiagen. No. 59104; USA) following the manufacturer’s recommendations. Different CpG methylation oligonucleotides were designed using Methyl Primer Express v1.0 software and the sequences are P1-F 5′-TTGGGTTTTGAGGAGT AGTA-3′, P1-R 5′-ACATCCTATTCCCTAATAAAAATT-3′; P2-F 5′-TATTGGTTTGGTATATATGGATGTA-3′, P2-R 5′-ATAAAACACTTAACTCRTACCRTA-3′; P3-F 5′- TTTGTGTAGTTGTGTTATGGTATATTT-3′, P3-R 5′- ACCCATAACAAACCACAACA-3′; P4-F 5′-TTGTGGTTTGTTATGGGTAAGTT-3′, P4-R 5′-TCAAAACATTCTCCATTAACAAAA-3′.

Each DNA sample was amplified by PCR as follows: 2.5 μl 10 × PCR buffer PCR reaction mix with 500 ng the bisulfite-treated DNA, 0.5 μl each of forward and reverse primers, 0.5 μl dNTP Mix, 0.5 μl rTaq (500 U, dNTP, Mg^2+^) (Takara Bio, Tokyo, Japan), addition of ddH_2_O up to a volume of 25 μl. After activation of polymerase at 94 °C for 10 min, it was followed by 40 cycles of the following sequence: 30 s at 94 °C, 30 s at 58 °C, 1 min at 72 °C, and final extension at 72 °C for 10 min.

### Cell Culture and Treatment

A549 cells were purchased from ATCC (Manassas, VA, USA) and were cultured in 1640 supplemented with 10% fetal bovine serum (FBS) at 37 °C and 5% CO_2_ in a humidified incubator. The cells were plated on 96-well plates, incubated with different concentrations of SiO_2_ NPs: 62.5, 125, 250, 500, 1000, and 2000 μg/ml for 24 h.

### Cell Viability Assay

Cellular viability was evaluated by the CCK8 proliferation assay. Cells were plated at a density of 1.5 × 10^4^ per well in a 96-well plate and incubated overnight. After exposure to SiO_2_ NPs at different concentrations, 100 μl of CCK8 was added to each well, and the cells were incubated for 30 min at 37 °C to allow CCK8 metabolism. At last, absorbance was determined at 450 nm. The cell-inhibiting rates were calculated and transformed into the IC_50_ using SPSS 15.0.

### Statistical Analysis

All computations were performed using the SPSS 15.0 software. Comparisons between groups were made using unrelated *t* tests and a Pearson chi-square test for BSP. The data are presented as the mean ± SD. In all cases, a value of *p* < 0.05 was considered statistically significant.

## Results

### Characterization of SiO_2_ NPs

**Fig. 1:**
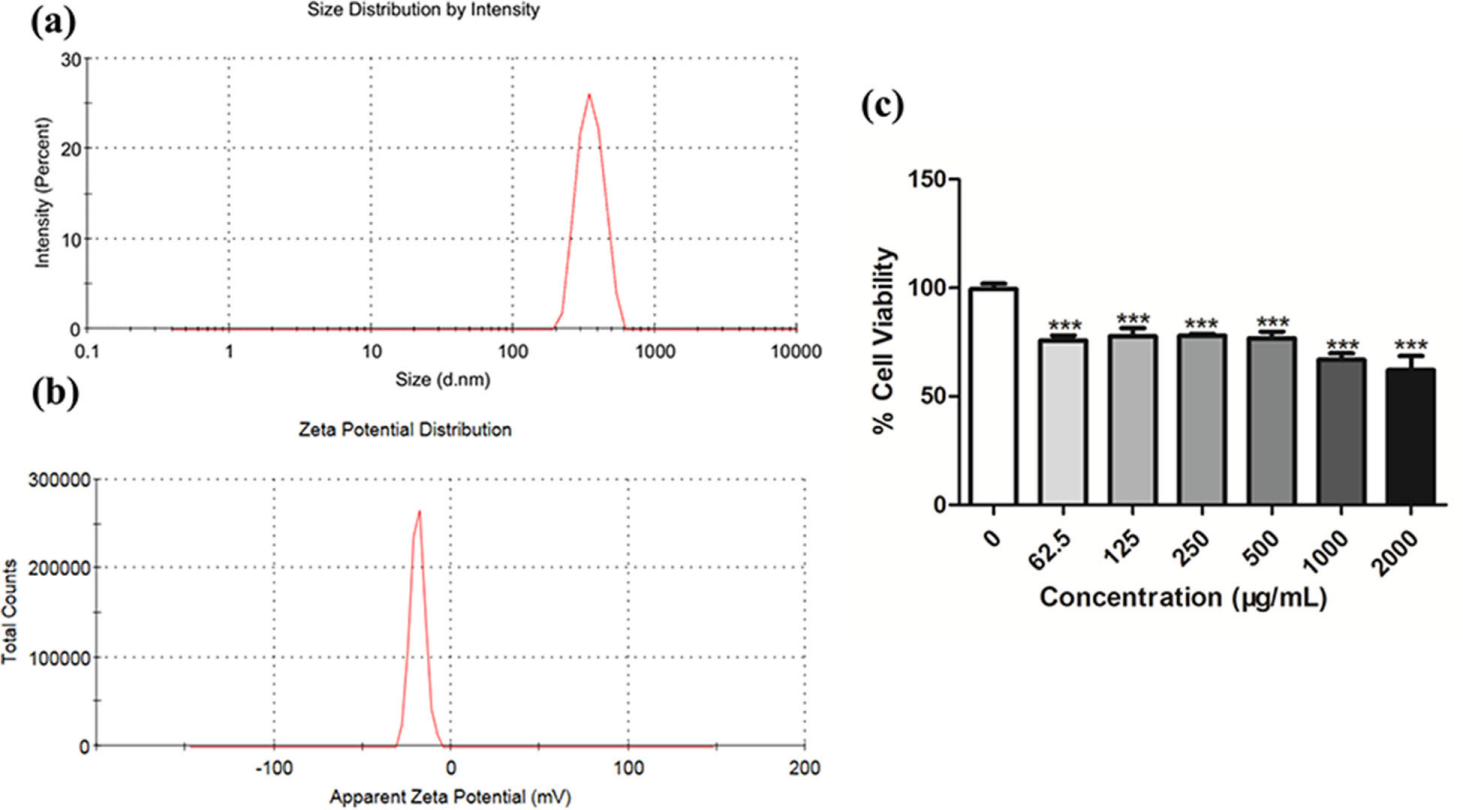
Characterization of SiO_2_ NPs in suspension. Particles were suspended in cell culture medium with 10% FBS. **a, b** Size and zeta potential of SiO_2_ NPs was assessed by Zetasizer Nano ZSP. **c** Cell viability was determined by CCK8 assay after exposure to various concentrations of SiO_2_ NPs for 24 h. Averages ± SD of duplicate experiments, each consisting of three technical replicates. *** *p* < 0.001

We characterized SiO_2_ NPs under experimental conditions. The average hydrodynamic radius and zeta potential of SiO_2_ NPs in culture medium were 371.77 ± 18.46 nm and 18.83 ± 2.12 mV, respectively (Fig. 1).

### Effect of SiO_2_ NPs on the A549 Cell Line

To determine the toxicity of SiO_2_ NPs, we performed a proliferation test with A549 cells, to determine the IC_50_ of SiO_2_ NPs on A549 cells. As illustrated in Fig. 1c, SiO_2_ NPs decrease A549 cell viability in a concentration-dependent manner. The reduction in cell viability is significant at SiO_2_ NP concentration of higher than 62.5 μg/ml (*p* < 0.001). The IC_50_ 24 h of a chemical is defined as a concentration that affects 50% of cell after 24 h of exposure. The IC_50_ 24 h determined for SiO_2_ NPs was 4942 μg/ml.

### Effects of SiO_2_ NPs on Murine Lung and Testis

**Fig. 2:**
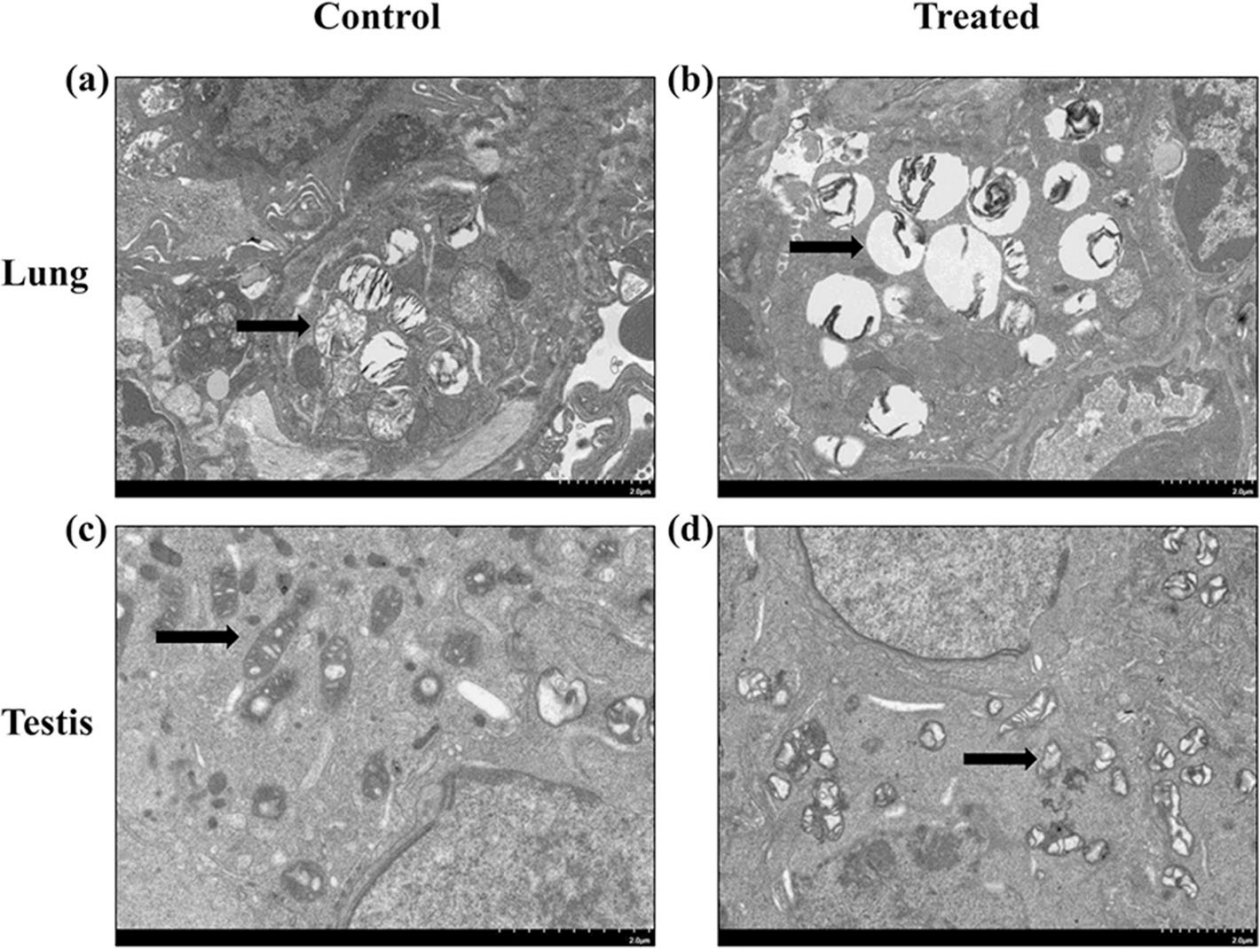
TEM images of lung and testis tissues of rats exposed to SiO_2_ NPs. **a** Morphology was assessed in lung tissues by SEM in controls. **b** Morphology was assessed in lung tissues by SEM in treated group. **c** Morphology was assessed in testis tissues by SEM in controls. **d** Morphology was assessed in testis tissues by SEM in treated group. Scale bars represent 2.0 μm

We determined whether exposure to SiO_2_ NPs at dose of 12.5 mg/kg body weight would lead to lung membrane damage and even testis damage in our mouse model. As shown in figures, SiO_2_ NP exposure led to disrupted lamellar body in histological sections of the lung (Fig. 2a, b) and a mitochondrial cristae damage in comparison to the control group in testis (Fig. 2c, d). We then investigated the lung and testis effects of SiO_2_ NPs on the activation of imprinting on the Dlk1/Dio3 imprinted region.

### Expression of the Imprinted Genes on the Murine Lung and Testis

**Fig. 3:**
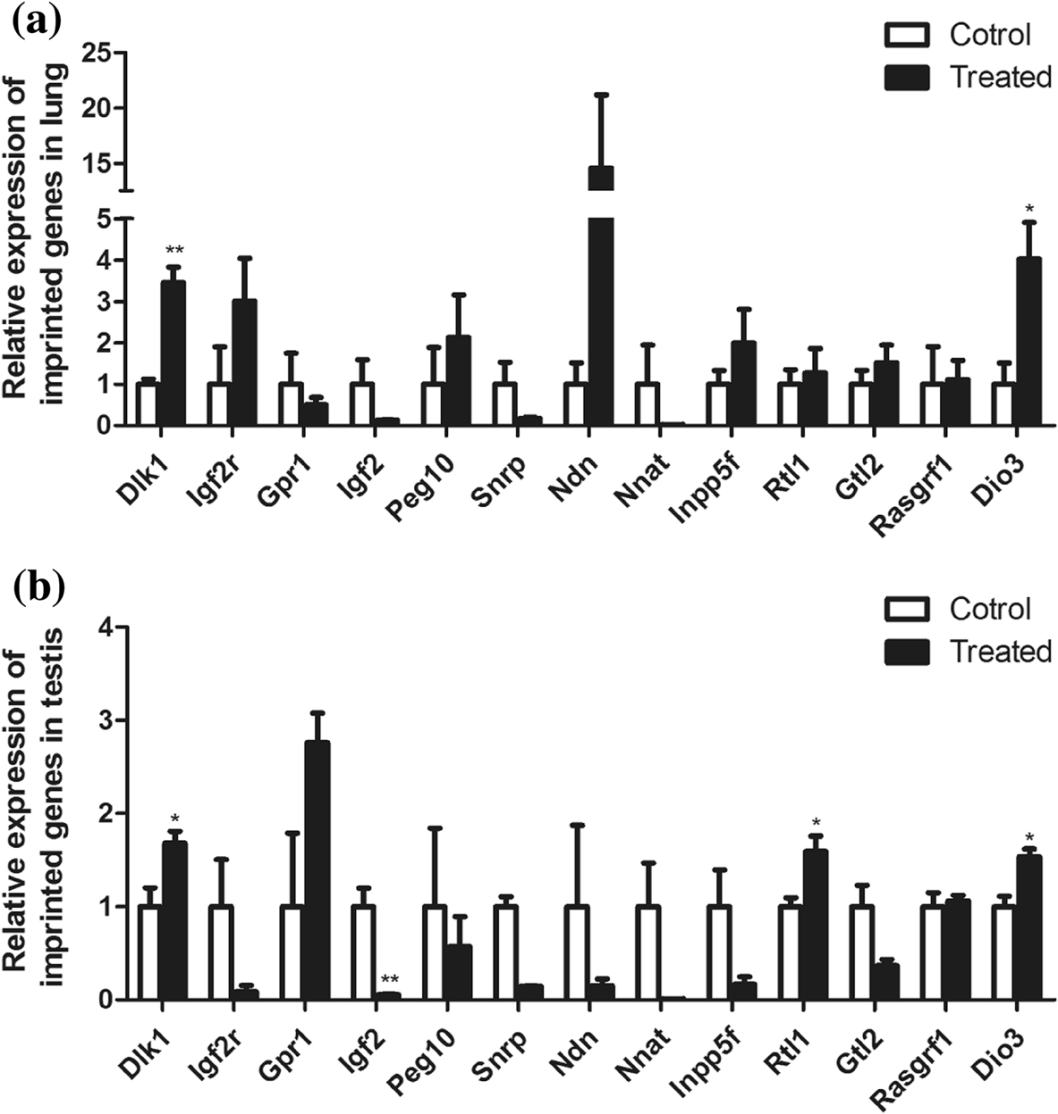
Expression of imprinted genes in lung and testis. **a** The expression of imprinted genes in lung. **b** The expression of imprinted genes in testis. *P < 0.05. **P < 0.01. Student’s *t* test

In order to illustrate the changes in lung and testis, we detected the imprinted genes in these tissues. We choose the 24 imprinted genes; they were *Dio3*, *Ddc*, *Dlk1*, *Gpr1*, *Gtl2*, *H19*, *Igf2, Igf2as*, *Igf2r*, *Inpp5f*, *Magel2*, *Magi2*, *Mest*, *Mir296*, *Mir298*, *Ndn*, *Nnat*, *Peg10*, *Plagl1*, *Pwcr1*, *Rasgrf1*, *Rtl1*, *Snrpn*, and *Snurf.* Thirteen of these genes are expressed in both lung and testis: *Dio3*, *Dlk1*, *Gpr1*, *Gtl2*, *Igf2r*, *Igf2*, *Inpp5f*, *Peg10, Ndn*, *Nnat*, *Rasgrf1*, *Rtl1*, and *Snrpn* (Fig. 3a, b). The differentially expressed genes of primary focus were on the Dlk1/Dio3 imprinted region, which contains *Dlk1*, *Gtl2*, *Rtl1*, and *Dio3*.

### Expression of the Dlk1/Dio3 Imprinted Region

**Fig. 4:**
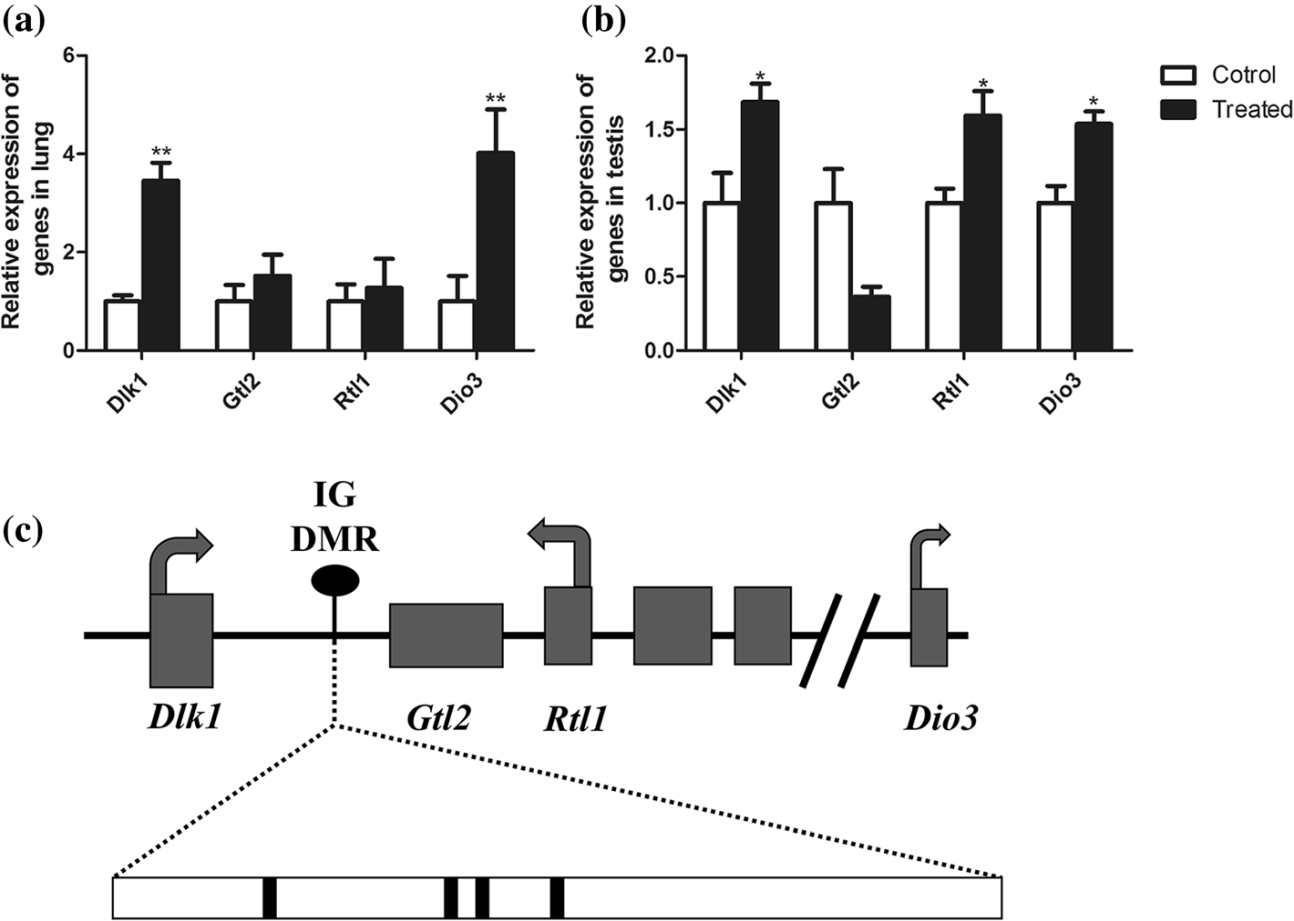
The expression of Dlk1/Dio3 imprinted region. **a** The Dlk1/Dio3 region genes expressed in the lung. **b** The Dlk1/Dio3 region genes expressed in the testis. **c** Schema of the Dlk1/Dio3 region. *P < 0.05. **P < 0.01. Student’s *t* test

The imprinted Dlk1/Dio3 region contains three protein-coding genes (*Dlk1*, *Gtl2*, *Rtl1*, and *Dio3*) on the inherited allele [20] (Fig. 4c). To elucidate the role of the Dlk1/Dio3 region in lung and testis tissue response to SiO_2_ NP treatment, we analyzed the methylation pattern of DMR compared with the controls. Different genes are targeted by methylation in the lung and the testis. The expression of the *Dlk1* and *Dio3* were upregulated in both the lungs and testis, while *Rtl1* was upregulated only in testis (Fig. 4a, b).

### The Methylation of Dlk1/Dio3 DMR Regions

**Fig. 5:**
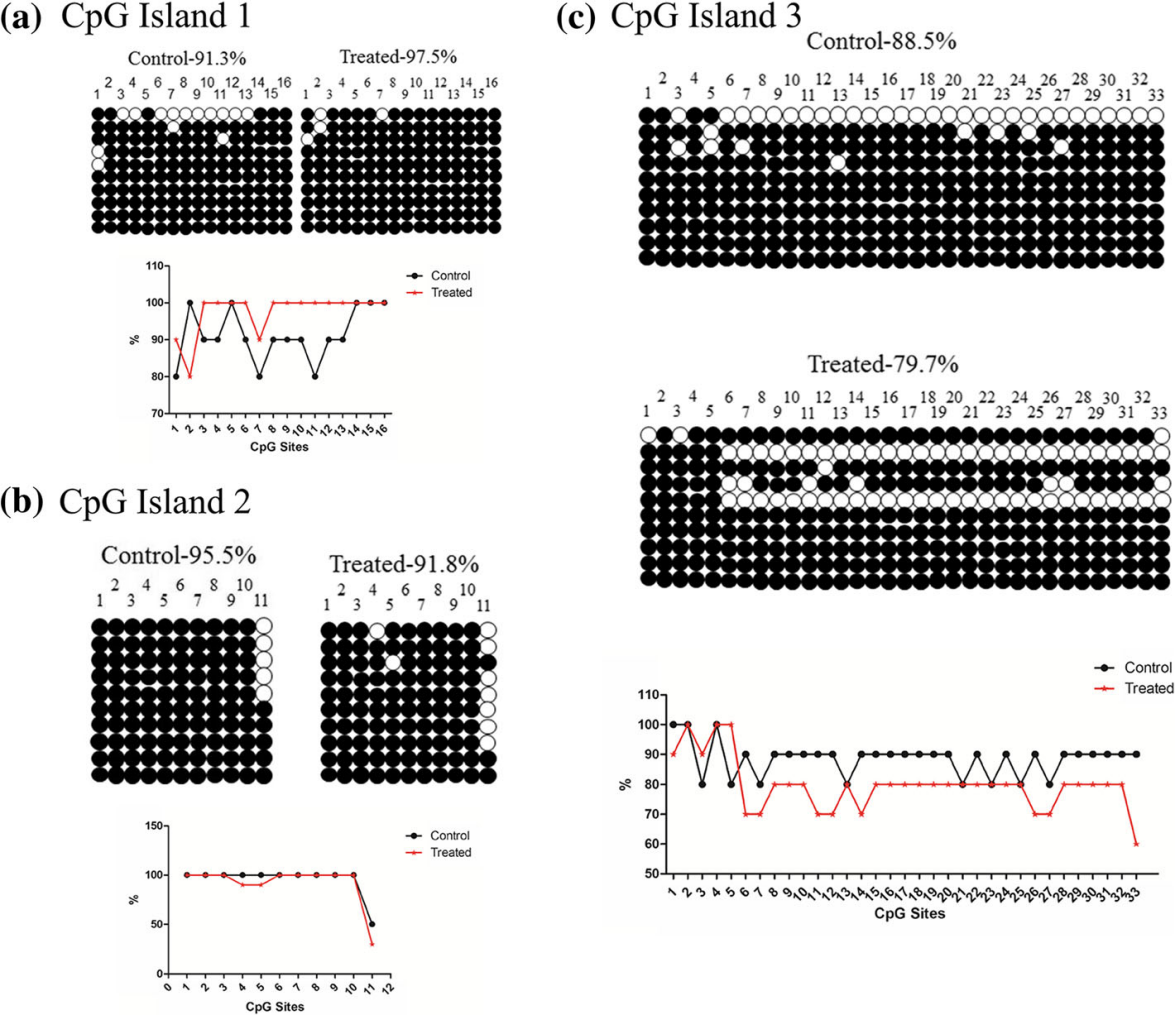
The methylation of Dlk1/Dio3 DMR regions in testis. **a** The methylation of CpG island 1 in the control and treated tissues. **b** The methylation of CpG island 2 in the control and treated tissues. **c** The methylation of CpG island 3 in the control and treated tissues

**Fig. 6:**
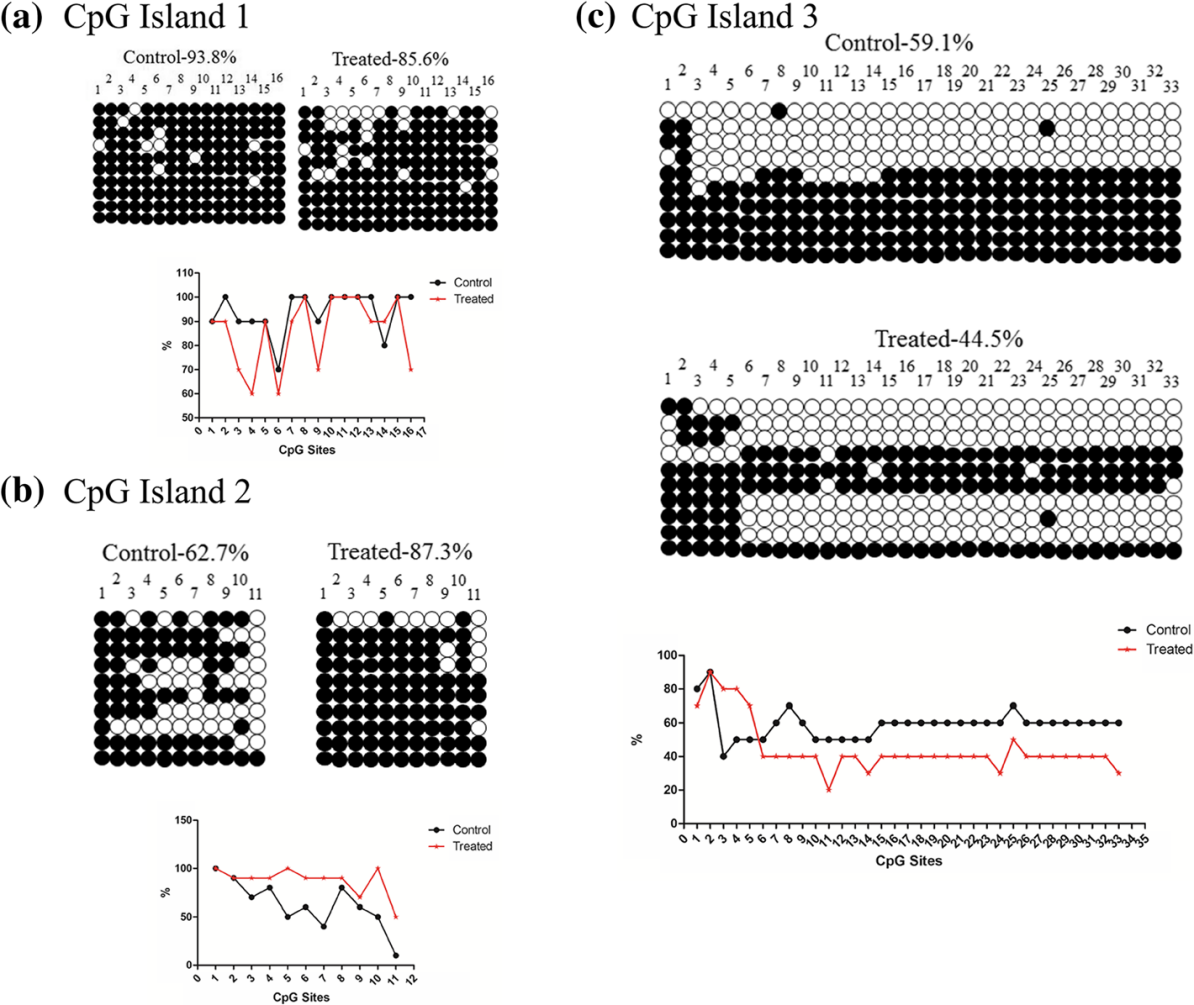
The methylation of Dlk1/Dio3 DMR regions in lung. **a** The methylation of CpG island 1 in the control and treated tissues. **b** The methylation of CpG island 2 in the control and treated tissues. **c** The methylation of CpG island 3 in the control and treated tissues

To further investigate whether the expression of genes changes in response to DNA methylation, we addressed the methylation status of this region in the mouse lung and testis. In DNA methylation analysis, we determined the sequences of the three sections of CpG islands. In the testis, they are hypomethylated; however, in CpG island 1, they are significantly hypermethylated (Fig. 5). In the lung, the whole methylation is the same as in the testis, while the CpG island 2 showed hypermethylation (Fig. 6).

## Discussion

The increasing use of nanomaterials has raised concerns about potential impacts on human health and environmental impacts. Previous studies have demonstrated that SiO_2_ NPs can cause lung and cardiovascular damage, such as lung inflammation and myocardial ischemic damage in old rats [21]. Furthermore, nanoparticles may have an effect on germlines, as such cells appeared to be more sensitive to the toxic effects of Ag NPs and demonstrated adverse effects following exposure to lower doses. Ag NP exposure increased the number of abnormalities observed in rat spermatic cells and reduced the integrity of both the acrosome and plasma membrane in addition to reducing mitochondrial activity [22]. Our investigation is part of a series of studies using an experimental platform to evaluate the potential of nanoparticles to target male organisms and even their unexposed offspring.

In our previous in vitro study, we reported that short-term exposure to some nanoparticles results in cell apoptosis and aberrant expression of imprinted genes in TM-4 Sertoli cells. These findings demonstrate that abnormal expression of imprinted genes may be an underlying mechanism for nanoparticles to induce reproduction toxicity [23]. Furthermore, in our previous in vivo study, some environmental factors, such as endocrine disruptors, also promote a phenotype or disease state not only in the individual exposed but also in successive generations of progeny. Epimutations in the germline that become permanently programmed can allow transmission of epigenetic transgenerational phenotypes [19]. The aim of this study was to investigate changes made to the epigenetic state by SiO_2_ NP treatment in a murine model in order to lay the mechanistic foundation of male transgenerational effects.

Epigenetic state is a term used to define chemical modifications that occur within a genome without changing the DNA sequence [24]. Epigenetic mechanisms, including DNA methylation, imprinted genes, histone modifications, and non-coding RNA expression, can affect genomic function in an exogenous environment [25]. To our knowledge, our study is the first to investigate SiO_2_ NPs inducing lung and testis toxicity at the epigenetic level.

We first examined the acute toxicity of the SiO_2_ NPs in A549 cells, a human lung epithelial cell line. However, our findings in experimental mice revealed injury in laminar lung type II epithelial cells and testicular mitochondrial crest injury after contact with SiO_2_ NPs at environmental concentrations [26]. In order to better understand the mechanism of the lung and testis pathology, we expressed imprinted genes. Genomic imprinting refers to silencing of one parental allele in zygotes of gametes depending upon the parent of origin; this silencing occurs via epigenetic processes such as DNA methylation and/or histone modification [27]. Previous studies have shown that imprinted gene expression at the Dlk1/Dio3 domain is important for fetal growth [28], the timing of human puberty [29], and susceptibility to metabolic disease [30]. Studies have suggested that the IG-DMR dictates the allelic methylation status of the Gtl2 promoter DMR, which then controls gene expression across the entire Dlk1/Dio3 region [17]. The main function of this imprinted control region is to inherit germ cell-driven DNA methylation as a gametic signal, and later to maintain subsequent allele-specific DNA methylation patterns within somatic cells [31]. Our study demonstrated that SiO_2_ NPs induce changes to the expression of the Dlk1/Dio3 region both in lung and testis tissues. In the Dlk1/Dio3 region, the paternal expressed genes (*Dlk1*, *Rtl1*, and *Dio3*) are particularly abnormal compared with the controls after NP treatment. Bisulfite sequencing results display different levels of hypomethylation in the lung and testis. The methylation state of IG-DMR is generally lower in treated tissues, and this hypomethylation may represent the mechanism of differential expression of imprinted genes.

## Conclusions

In conclusion, our results indicate that SiO_2_ NP exposure may induce important DNA methylation changes that trigger cellular damage and that these changes are highly important to the expression of the Dlk1/Dio3 imprinted gene cluster. Importantly, the changes in DNA methylation affect both the lung and testis tissues. These results play an important role in our future research examining the epigenomic effects of the nanoparticles inherited by offspring of exposed models and the clarification of the molecular mechanisms that mediate such epigenetic alterations.

## Additional file

Additional file 1: **Table S1.** List of 24 homologous imprinted genes. **Table S2.** List of primers used to test the expression of the 24 imprinted genes and the reference genes Gapdh and U6. (ZIP 22 kb)

## Abbreviations

BALF: Bronchoalveolar lavage fluids
CCK-8: Cell counting kit-8
DMRs: Differentially methylated regions
EGFR: Epidermal growth factor receptor
IG-DMR: Intergenic DMR
MPs: Microparticles
NPs: Nanoparticles
