# Supplementary material for: Effect of Nano-SiO2 on Expression and Aberrant Methylation of Imprinted Genes in Lung and Testis
Source: Nanoscale Res Lett. 2018 Sep 4;13:266. doi: 10.1186/s11671-018-2673-4 (PMC6123335; doi:10.1186/s11671-018-2673-4)
Supplement: Supplementary file 1 — Table S1. List of 24 homologous imprinted genes. Table S2. List of primers used to test the expression of the 24 imprinted genes and the reference genes Gapdh and U6. (ZIP 22 kb) [file 11671_2018_2673_MOESM1_ESM.zip › Table S2.docx]

**Table S2.** List of primers used to test the expression of the 24 imprinted genes and the reference genes *Gapdh* and *U6.*

| **Gene** | **Primer-F (5’-3’)** | **Primer-R (5’-3’)** |
| --- | --- | --- |
| *Ddc* | TGGGGACCACAACATGCTG | TCAGGGCAGATGAATGCACTG |
| *Dlk1* | AGCTGCACCCCCAACC | CTGCTGGCGCAGTTGGTC |
| *Dio3* | ATGCGTATCAGACGACAACCG | CCTTGTGCGTAGTCGAGGAT |
| *Gpr1* | GCTGGGAGTTGTTCACTGGG | GACGATGGCATTTCCTGGAAT |
| *Gtl2* | GTTTCTGGACTGTGGGCTGT | CAACAGCAACAAAACTCAGAACATTCA |
| *H19* | GCACCTTGGACATCTGGAGT | TTCTTTCCAGCCCTAGCTCA |
| *Igf2* | AGCCGTGGCATCGTTGAG | GACTGCTTCCAGGTGTCATATTG |
| *Igf2as* | TCTTTGCCCTCTTTCGTCTC | CTCCAGGTGCTTCCGTCTAG |
| *Igf2r* | CTGCCGCTATGAAATTGAGTGG | CGCCGCTCAGAGAACAAGTT |
| *Inpp5f* | ACTGAACCTGAGCAGATTTCCA | CCACCCCACTCCAAAAGGTT |
| *Magel2* | GGGCTCCGCTAAATCATTG | CCCCTGCGGTCTATAGAAGA |
| *Magi2* | GGACTAGCAGGGTTCACGAA | GCTCCGACGTACGGAAACT |
| *Mest* | TGACCACATTAGCCACTATCCA | CCTGCTGGCTTCTTCCTATACA |
| *Mir296* | ACACTCCAGCTGGGAGGGCCCCCCCTCAA | CTCAACTGGTGTCGTGGAGTCGGCAATTCAGTTGAGACAGGATT |
| *Mir298* | ACACTCCAGCTGGGAGCAGAAGCAGGGAGGTT | CTCAACTGGTGTCGTGGAGTCGGCAATTCAGTTGAGTGGGAGAA |
| *Ndn* | CTGATGATGTGTGTTGGGGTA | GGCTTTGCTGGTGACTTCTT |
| *Nnat* | GTACATCTTCCGCGTGCTG | CTTCTCGCAATGGGCTGT |
| *Peg10* | AAATTGCCTGACATGAAGAGGAGTCTA | AAGCCTAGTCACCACTTCAAAACACACTAAA |
| *Plagl1* | CATATTTGCATGTTAGAAGAATCAGC | TGAGTCAGTTAGGTCAGTGTAGAGAGA |
| *Pwcr1* | TGCTCTTTAGCAGGATGGTGT | GATGCAGGGATGCTCGTC |
| *Rasgrf1* | GCCAGAAGACTTGACAACGCT | TCAATCTACAGGGATGGTGGAAG |
| *Rtl1* | CCCCTGTGAGGCATCATCTAA | GAACCATCACTCGACTCCTGG |
| *Snrpn* | GAGGAGTTGGGGGACCAT | CAGCTGCTACAGTGCCTCTTC |
| *Snurf* | TCTGATTCCAAGCAAAAACCA | GTTCTCCTAGAAACAATGCAAGC |
| *Gapdh* | GCACCGTCAAGGCTGAGAAC | GGATCTCGCTCCTGGAAGATG |
| *U6* | CTCGCTTCGGCAGCACA | AACGCTTCACGAATTTGCGT |
| URP | TGGTGTCGTGGAGTCG |  |
|  |  |  |
