# Supplementary material for: Effect of Nano-SiO2 on Expression and Aberrant Methylation of Imprinted Genes in Lung and Testis
Source: Nanoscale Res Lett. 2018 Sep 4;13:266. doi: 10.1186/s11671-018-2673-4 (PMC6123335; doi:10.1186/s11671-018-2673-4)
Supplement: Supplementary file 1 — Table S1. List of 24 homologous imprinted genes. Table S2. List of primers used to test the expression of the 24 imprinted genes and the reference genes Gapdh and U6. (ZIP 22 kb) [file 11671_2018_2673_MOESM1_ESM.zip › Table S1.doc]

**Table S1.** List of 24 homologous imprinted genes.

| **Gene** | **Location** | | **Full Name** | **Gene Type** | **Expressed Allele** |
| --- | --- | --- | --- | --- | --- |
|  | **Mouse** |  |  |  |  |
| *Ddc* | 11 7.0 cM AS |  | dopa decarboxylase | PC | P |
| *Dio3* | 12; 12 F1 |  | deiodinase, iodothyronine type III | PC | P |
| *Dlk1* | 12 54.0 cM |  | delta-like 1 homolog (Drosophila) | PC | P |
| *Gpr1* | 1 AS |  | G protein-coupled receptor 1 | PC | P |
| *Gtl2* | 12 54.0 cM |  | gene trap locus 2 | NC | M |
| *H19* | 7 69.03 cM AS |  | H19, imprinted maternally expressed transcript | NC | M |
| *Igf2* | 7 69.09 cM AS |  | insulin-like growth factor 2 | PC | P |
| *Igf2as* | 7 69.09 cM |  | insulin-like growth factor 2, antisense | NC | P |
| *Igf2r* | 17 7.35 cM AS |  | insulin-like growth factor 2 receptor | PC | M |
| *Inpp5f* | 7 F3 |  | inositol polyphosphate-5-phosphatase F | PC | P |
| *Magel2* | 7 28.0 cM |  | melanoma antigen, family L, 2 | PC | P |
| *Magi2* | 5 |  | membrane associated guanylate kinase, WW and PDZ domain containing 2 | PC | P |
| *Mest* | 6 7.5 cM |  | mesoderm specific transcript | PC | P |
| *Mir296* | 2 AS |  | microRNA 296 | NC | P |
| *Mir298* | 2 AS |  | microRNA 298 | NC | P |
| *Ndn* | 7 28.0 cM |  | necdin | PC | P |
| *Nnat* | 2 88.0 cM |  | neuronatin | PC | P |
| *Peg10* | 6 0.5 cM |  | paternally expressed 10 | PC | P |
| *Plagl1* | 10 15.0 cM |  | pleiomorphic adenoma gene-like 1 | PC | P |
| *Pwcr1* | 7 29.0 cM AS |  | Prader-Willi syndrome chromosome region 1 | NC | P |
| *Rasgrf1* | 9 47.31 cM |  | RAS protein-specific guanine nucleotide-releasing factor 1 |  | P |
| *Rtl1* | 12 12 F1 |  | retrotransposon Gaglike 1 | PC | P |
| *Snrpn* | 7 29.0 cM AS |  | small nuclear ribonucleoprotein N | PC | P |
| *Snurf* | 7; 7 B5 |  | surfeit gene cluster | PC | P |

Note: PC, protein coding; NC, non-coding; P, Paternal; M, Maternal
